# EURAPA moves to open access: Research trends and challenges in physical activity in old age

**DOI:** 10.1186/s11556-015-0149-4

**Published:** 2015-10-07

**Authors:** Yael Netz, Wiebren Zijlstra

**Affiliations:** The European Group for Research into Elderly and Physical Activity, Wingate College, Wingate Institute, Netanya, 4290200 Israel; Institute of Movement and Sport Gerontology, German Sport University, Cologne, 50933 Germany

The rise of the Internet has radically changed scholarly communication. It is now widely recognized that having an open access to research results contributes to better and more efficient science, and to innovation in the public and private sectors [1]. The global trend towards offering free online (open) access to the results of publicly-funded research (publications and data) has been a core strategy in the European Commission to accelerate the circulation of knowledge and thus innovation [1]. In accordance of this trend, the European Union (EU) is striving to improve access to scientific information and to boost the benefits of public investment in the research funded under the EU framework [2] so that everyone with access to the Internet can benefit from the latest scientific insights.

As Editors-in-Chief of *EURAPA*, we very much look forward to the changes entailed in the journal’s transition to BioMed Central. Thanks to the open access format, the quality research and review articles that appear in our journal will be available to a much larger audience with an interest in aging and physical activity than was possible until now.

*EURAPA* is the official publication of the European Group for Research into Elderly and Physical Activity (EGREPA). EGREPA fills an important niche in the study of physical activity for the elderly. The first volume of *EURAPA*, as the official journal of EGREPA, was issued on the occasion of the 6th World Congress on Aging and Physical Activity in London, Ontario, Canada in 2004. The first volumes were published without a publisher. The decision to publish *EURAPA* with Springer Publishers was taken in 2005. The first issue with Springer, Volume 3, Number 1, appeared in May 2006. *EURAPA* has meanwhile become a well-established and accepted peer-reviewed journal. With Volume 8, Number 1, 2011, *EURAPA* became an electronic-only version. The year 2011 also marked the introduction of new publication formats so that *EURAPA* began to accept papers in categories such as: original research, theoretical and methodological articles, brief reports, guest editors and guest editorials [3].

In January 2015 the journal began publishing with BMC with the intention of further expanding its role as an interdisciplinary platform for the development and dissemination of scientific research relating to physical activity and aging. A new editorial board, comprised of new scientists in all relevant areas was created. This is a great opportunity to express our appreciation and thanks to the founders of the journal – Prof. Michael Sagiv and Prof. Heinz Mechling. Prof. Sagiv was the Co-Editor-in-Chief for life and medical sciences and Prof. Mechling for behavioral sciences. Both of them were pioneers in research on physical activity and aging and contributed greatly to the success of the journal. We also extend our thanks to Dr. Michael Brach, who has served as an associate editor and continues to contribute in making the journal an important voice for research in our field.

We would also like to extend sincere thanks to three academic institutes that have provided essential resources and necessary operational support to both EGREPA and *EURAPA*: The Zinman College of Physical Education and Sport Sciences at the Wingate Institute; The Institute of Movement and Sport Gerontology at the German Sport University, Cologne (former at University of Bonn); and the Institute of Sport at the Westfälische Wilhelms-University, Munster. We hope these institutes continue to provide their support. We also take the opportunity to thank all of our past reviewers for their service to the journal, the authors for their willingness to publish their work in *EURAPA*, and the readers, who have accepted *EURAPA* as a part of the scholarly literature.

## Research trends and challenges in physical activity in old age

While there is sufficient evidence to assume the benefit of physical activity across the human lifespan [4, 5], many questions regarding physical activity in old age remain unanswered. These questions encompass basic philosophical issues related to aging and/or movement, questions seeking to examine the underpinnings of movement, and specific questions related to unique aspects of physical activity and movement in advanced age. Starting from broad philosophical themes and then honing in on more specific issues, we present here our selection of issues which are currently in the forefront of research about physical activity in old age and which will probably continue to constitute central research topics in the near future.

### Research on movement and mobility – reversing the aging process or improving quality of life?

A recent editorial focusing on aging as a science raised a philosophical question: “*Is aging a failure or a conquest of natural selection?*” ([6], p.2). “*If aging is a failure*”, it was said, *“it is then only the accumulation of disparate damage and the control of aging is, therefore, achievable only in part and at the cost of enormous effort”* ([6], p.2). The implications for research on physical activity are that we should assess physical activity programs only in terms of their ability to improve performance and functioning and to attenuate or slow down the aging process. *“Conversely, if aging is a conquest of natural selection, thus genetically programmed, it means that we can envisage the possibility that the phenomenon may be brought entirely under our control”* ([6], p.2). The implications for research in physical activity are that we should explore the genetic program of movement to fully control the aging process of the movement system. In effect, these two theories co-exist simultaneously in the area of physical activity, with one line of research looking into the benefits of physical activity in terms of performance and functioning while the other strives to discover the genetic, molecular and cellular mechanisms underlying movement.

### Physical activity in old age – a need or a challenge to human nature?

Movement is a basic need of any living creature including humans, at the same time, human nature is regulated by the *principle of economy* – the aspiration to reduce physical and mental efforts to a minimum. These principles also underlie the development of cutting-edge technology (e.g. transportation, communication, home school and work environment) which has actually caused a decrease in physical activity in all ages and led to the field of “sedentary physiology” [7]. This direction of research must grapple with a cardinal question: Is it possible that our efforts to promote physical activity (given its benefits) are in fact in competition with and a challenge to human nature? If this is the case, what are the implications for designing effective programs for enhancing physical activity?

This struggle with human nature is reinforced when investigating advanced age. There is a natural decline in physical activity with age that seems to have a biological basis in nonhuman subjects [8] and humans [9]. The dopaminergic neurotransmitter system appears to be one possible neurobiological mechanism that explains this decline [8]. Interestingly, this mechanism not only affects the ability to move, but also involves motivational factors or the “will” to participate in physical activity. As a result, sedentary behavior has become a focal point of research independent of physical activity [10, 11] and an increasing effort has been invested in the development of innovative strategies such as physical exercise software based on motion tracking (exergaming), remote coaching, mobile applications etc. for encouraging older adults to engage in exercise [12, 13]. At the same time there are continuous attempts to understand people’s motivation and beliefs regarding physical activity, including the use of qualitative techniques such as depth and unstructured interviews [14]. Raising awareness of the benefits, minimizing the perceived risks of physical activity, and improving the environmental and financial access to physical activity opportunities are typical recommendations for changing exercise behavior [14].

### Exercise and behavior – what is the cause and what is the effect?

The view of physical activity as an independent vs dependent variable in research is a fundamental research issue. Regarding the role of the dopaminergic system in regulating voluntary physical activity behavior, it has been asked whether physical activity induces changes in neurotransmitter systems, thus making physical activity an independent variable, or whether the opposite is true: the dopaminergic system has a regulatory role on physical activity, thus making physical activity a dependent variable [15]. Apparently there is evidence supporting both hypotheses, and a dual role has been proposed. Physical activity produces beneficial changes in the dopamine system including increased dopamine signaling as well as increased BDNF levels in the brain, and in this role, physical activity is an independent variable. But dopamine has a role in motivation for natural rewards, thus controlling the “wanting” and/or motivation for physical activity – thus making physical activity a dependent variable [15].

### Examining the underpinnings of human movement

This reciprocal causation between behavior (physical activity) and the biological mechanisms which underlie movement behavior goes far beyond the willingness to exercise. Physical activity and depression is another example of the interplay between genetics and behavior currently addressed in the research [16].

The inquisitiveness of human nature along with the aspiration to bring the human body and the aging phenomenon entirely under our control [e.g. 6] have led researchers to probe deeply into the cellular, molecular and genetic mechanisms of movement. The causal processes of development, with an emphasis on interactions among genes and between genes (nature) and the environment (nurture) is the study of epigenetics [17]. Modern epigenomics helps to end nature-nurture debates as it is now possible to measure the interface between genes and the environment directly [17]. This measurement will have ramifications for health and human disease due to an ageing and an increasingly physically inactive population [17].

Of special interest is research on the effect of physical activity on telomere length, which is considered to be a marker for the rate of aging. A recent review on this topic came to the equivocal conclusion that a possible significant association between physical activity and telomere length remains an open question [18], which means that this will continue to be an interesting area of research in the near future.

### Exercise and health - dose–response relationship?

While there is a general agreement that exercise is necessary for optimal health, one issue explored in that respect and probably will be explored in the future is the dose–response relationship between exercise and health [19, 20]. How much exercise is best for health? “No pain no gain”? No pain big gains”? There is generally an agreement that moderate exercise is sufficient for optimal health [19], on the other hand there is also evidence that exercising more than the recommended dose may have additional benefits [19] and that compared with the traditional moderate-intensity continuous exercise training, high-intensity interval exercise may have superior metabolic, cardiac, and systemic vascular adaptation [20]. Unravelling dose–response relationships in advanced age will be a challenge especially in chronic diseases since exercise cannot be as easily administered as pharmaceutical interventions.

### Musculo-skeletal issues

The musculo-skeletal system as related to the decline in human muscle mass and strength (sarcopenia) is another track of research. Recent reviews on this topic point out the key variables that should be investigated in the effort to maximize the antiaging effects of exercise. One review [21] elaborates on mitochondrial dynamics in aging skeletal muscle, concluding that the different effects of aerobic vs resistance exercise in improving mitochondrial content and quality point to the advantage of tailoring combined physical activity programs on an individual basis. Other reviews suggest investigating dietary strategies [22] and myofilament adaptations [23].

### Exercise and chronic diseases

The desire to better understand the mechanism mediating between exercise and chronic diseases such as cardiovascular disease, diabetes, and some types of cancers is another focus of research. Exercise and gene expression in human white blood cells in relation to inflammation and atherosclerosis was the topic of a recent systematic review aimed at understanding the molecular mechanisms involved in the relationship between physical activity and reduced risk of these chronic diseases [24]. Yet another review concentrated specifically on the effect of exercise on people at high cardiovascular risk [25]. The conclusion of this review was also indefinite. Serious methodological limitations in current research make it impossible to elicit any clear-cut conclusions. Two other reviews reported on the relationship between physical activity and chronic disease. One showed that reducing sedentary time can improve lipid and glucose metabolism for the prevention and treatment of type 2 diabetes [26], and the other reported on an association between physical activity and reduced risk of some types of cancer [27]. It is likely that the relationship between physical activity and chronic diseases will continue to serve as a focus of exploration in terms of the cellular, molecular and genetic mechanisms involved in this relationship. At the same time, researchers will also continue to seek the most effective physical activity programs for attenuating the aging decline and optimizing function and performance under the irreversible losses caused by chronic diseases. A better understanding of the mechanisms will help developing more effective physical activity programs.

### Brain and cognition as related to physical activity

Brain and cognition in advanced age have been at the forefront of research [28], and brain plasticity [29] as well as cognitive plasticity [30] have been heavily researched and will probably continue to be researched. While findings of studies seem to be promising, some studies seriously question the effect of physical activity on cognition in advanced age [31], and others report only limited data on this effect [32]. At the same time, the effect of physical activity on pathologies of the CNS such as Alzheimer’s disease, depression, and Parkinson’s disease has also received a lot of attention in recent research. The findings of a recent review suggest that the regional physiological adaptations that occur with exercise could constitute a promising research field leading to the recovery of psychiatric and neurological health conditions [33]. Alzheimer’s disease has been of special interest and the conclusions are that more studies, exploring the relationship between physical fitness and brain neuroplasticity [34], as well as studies assessing the effect of physical activity on performance and functioning of the patients [35], are needed.

### Motor control

Motor control and aging, examined both by neuroimaging techniques as well as by performance tasks, is a stimulating path of research. In a review focusing on motor inhibition it was concluded that a promising direction for future research would be to correlate individual differences in structural and/or functional integrity of principal brain networks with observed changes in inhibitory processes within cortico-cortical, interhemispheric, and/or corticospinal pathways [36]. One interesting direction of research might be to link these changes with other typical changes of the aging CNS, for example to explore the relationship between motor inhibition and cognitive inhibition controlled by the prefrontal cortex. In view of the working hypothesis that age-related deficits in motor functions can be explained by deficits in cognitive functions, the question emerges whether it may be possible to alleviate both cognitive and motor inhibitory functions by promoting an active life style.

### Mobility, Balance and gait

These themes constitute the focus of many studies, due to their key role for independent functioning. A review of neuroimaging studies of mobility in aging explored the relationship between mobility and the structure and function of the aging brain, and found that mobility was associated with reduced gray and white matter volume and with activation patterns of the cerebellum, basal ganglia, parietal and frontal cortices. Increased involvement of the prefrontal cortex was evident in both imagined walking conditions and conditions where the cognitive demands of locomotion were increased [37].

The effectiveness of physical activity programs in improving balance is not always straightforward. One review described an association between trunk muscle strength and balance, and improvement in trunk muscle strength and in balance as a result of core strength training [38], and another review reported improvement in balance and mobility as a result of individualized home based exercise [39]. Other reviews, however, have yielded limited findings. Whole body vibration improved selected balance and mobility measures but its impact remained inconclusive [40], and methodological limitations made it impossible to evaluate the potential of Wii-based exercise for improving balance [41]. Likewise, no clear effect of resistance exercise on gait and balance was observed in Parkinson’s disease patients [42].

### Mindful activities

On the other hand, the reports on the effect of Pilates on balance seem to be more encouraging. Two reviews indicated improved balance following Pilates [38, 43], and additional review reported improvements in balance, walking and gait performance as well as improvements in other aspects of physical and emotional functioning [44]. In light of these results, and some other indications regarding the benefits of Tai-Chi to the aging cognitive system, as mentioned in previously mentioned reviews [28, 32], it is expected that Pilates, Tai-Chi and other mindful activities will probably attract researchers in aging.

### Falls prevention

Fall prevention is an essential issue in old age, and the potential of physical activity to prevent falls has been and probably will be heavily researched. While previous reviews reported that exercise as a single intervention can prevent falls [45, 46], current findings are inconclusive. A review on cognitive-motor interventions reported improvements on various fall risk factors such as balance, strength, attention and executive functions [47], and as was previously described a review on Pilates informed improved balance [43]. Both reviews, however, claimed that the effect of these interventions on falls has not been definitively demonstrated. An interesting review elaborating on fear of falling [48] concluded that exercise interventions such as Tai Chi, yoga, balance or strength training probably reduce fear of falling immediately after the intervention, but it is not clear whether the interventions reduce fear of falling beyond the end of the intervention or whether they affect other outcomes such as risk of falling, depression or the amount of physical activity.

### Mobility, movement performance, and the brain as related to exercise interventions

Taken together, there is growing evidence regarding the relationship between mobility and the aging brain – in terms of both structure and functioning [37]. Furthermore, emerging findings elucidate cellular and molecular mechanisms by which exercise and energy intake modify the plasticity of neural circuits in ways that affect brain health [29]. Nonetheless, this information has not yet been translated into effective exercise programs for improving mobility. Clearly, there are many aging-related changes in the brain which may affect mobility and more insight is needed in the contributions of different central processes in order to design effective exercise programs for older adults. Thus, studies investigating the relationship between specific brain structure and functioning with exercise performance will form another body of research. It is expected that increasing numbers of studies will use multimodal strategies for exploring brain structure and functioning as related to movement performance, and multi-domain synergistic interventions including various exercise modes as well as other interventions [e.g. Ballesteros] for improving quality of life in old age.

### What this means for *EURAPA*

And finally, referring to Libertini’s two hypotheses mentioned in the beginning of this article, he postulated that “*the theoretical arguments and empirical data that support or refute the two hypotheses must be carefully evaluated, without any uncritical acceptance of either of the two alternatives*” ([6], p. 2). As editors of *EURAPA*, we want to promote both directions of research. While on the one hand, we welcome research that explores possible alteration of the genetic programing of movement for the purpose of reversing the aging process, we also encourage researchers to examine, modify and improve physical activity programs for improving quality of life, based on the hypothesis that aging is an irreversible process of gradual and not so gradual loss of function.
